# Shear force modulates the activity of acid-sensing ion channels at low pH or in the presence of non-proton ligands

**DOI:** 10.1038/s41598-019-43097-7

**Published:** 2019-05-01

**Authors:** Daniel Barth, Martin Fronius

**Affiliations:** Department of Physiology and HeartOtago, University of Otago, Dunedin, New Zealand

**Keywords:** Ion transport, Ion channel signalling

## Abstract

Acid-sensing ion channels (ASICs) belong to the degenerin/epithelial sodium channel protein family that form mechanosensitive ion channels. Evidence as to whether or not ASICs activity is directly modulated by mechanical force is lacking. Human ASICs (hASIC1_V3_, hASIC2a and hASIC3a) were heterologously expressed as homomeric channels in *Xenopus* oocytes and two-electrode voltage-clamp recordings were performed. hASIC3a was expressed in HEK-293 cells and currents measured by whole-cell patch-clamp recordings. ASIC currents in response to shear force (SF) were measured at pH 7.4, acidic pH, or in the presence of non-proton ligands at pH 7.4. SF was applied via a fluid stream generated through a pressurized perfusion system. No effect was observed at pH 7.4. Increased transient currents for each homomeric channel were observed when elevated SF was applied in conjunction with acidic pH (6.0–4.0). The sustained current was not (hASIC2a) or only slightly increased (hASIC1_V3_ and hASIC3a). SF-induced effects were not seen in water injected oocytes and were blocked by amiloride. Non-proton ligands activated a persistent current in hASIC1_V3_ and cASIC1 (MitTx) and hASIC3a (GMQ) at pH 7.4. Here SF caused a further current increase. Results suggest that ASICs do have an intrinsic ability to respond to mechanical force, supporting their role as mechanosensors in certain local environments.

## Introduction

Acid-sensing ion channels (ASICs), formed by ASIC proteins, are members of the degenerin/epithelial Na^+^ channel protein superfamily^1^. Members of this protein family were initially identified to form mechanoreceptors in *Caenorhabditis elegans*^2^ and it is considered that their mechanosensitive function relies on an interaction with the extracellular matrix^3^.

ASICs form voltage-insensitive cationic channels that are usually activated by a drop in external pH. To date, five genes have been identified (*ASIC*1–5) that encode five subunits^1^ although for some subunits multiple isoforms/variants have been described. Each subunit contains two transmembrane domains, an extracellular loop and intracellular N- and C-termini and crystallographic studies on chicken ASIC1 (cASIC1) revealed that the functional channel assembles as a trimer^4^.

ASICs are expressed in both the central (CNS) and peripheral nervous systems (PNS). Differences between the expression of certain subunits with regard to their localization (CNS vs PNS) have been reported and that these expression patterns may be species dependent^5^.

Since most ASICs are activated by protons^6^, ASICs in the PNS are considered to play an important role in nociception particularly associated with a local drop in pH. It is suggested that these changes in local pH can occur during periods of high metabolic demand associated with inflammation^7^, tissue lesions, ischemia and tumours^8^. The drop in pH is suggested to activate ASICs localized in nerve endings in the periphery. This depolarizes the membrane potential, to initiate excitation of action potentials and to induce the perception of pain. In the CNS ASICs are suggested to play a role in seizure termination. Seizures are also associated with decreased pH due to high metabolism and local acidification from synaptic vesicle release^6^ that activates ASICs in neurons and contributes to the termination of seizures^9^. Further, there is emerging evidence that in addition to protons, non-proton ligands can activate ASICs at physiological pH^10^ and a recent study reported that changes of local membrane lipid environments can activate ASIC3, independent of changes of pH^11^. This is important since it indicates that protons may not be the sole trigger for activating ASICs. As non-proton ligands a peptide complex from the venom of the Texas coral snake (*Micrurus tener tener*, MitTx) was shown to activate ASIC1 at normal pH^12^ and 2-guanidine-4-methylquinazoline (GMQ), a small molecule that is similar to endogenously produced inflammatory mediators, was identified to activate ASIC3 at pH 7.4^13^ and modulates pH responses of ASIC1 and 2^14^.

Another function of ASICs – that is independent of their role as pH sensors – has been proposed as mechanosensors^15–18^. In accordance with this role ASIC subunits were detected in cutaneous mechanosensitive receptors including the Meissner corpuscles, Merkel cell neurite complexes and lanceolate nerve endings^18,19^. ASIC1 is further localized in dorsal root ganglia that innervate the colon and are suggested to contribute to visceral mechanosensation^20^. Another aspect for a mechanosensitive role of ASICs is indicated by the detection of ASIC1, 2 and 3 subunits in nerve terminals of the aortic arch^21^ and ASIC2 contributing to myogenic constriction of renal vessels^22^, suggesting a role in blood pressure regulation. Other examples, for a potential involvement in mechanosensation, derive from their localization in human bladder (mediating pain perception in bladder pain syndrome)^23^ and fish gill epithelium where ASICs would be constantly exposed to shear force (SF) due to the water flowing over the surface of the gill epithelium^24^.

More striking, but also confusing evidence about ASICs potential role as mechanosensors derives from a series of experiments performed with various transgenic animals. Recent overviews about the outcome of these studies are extensively reviewed in two articles^19,25^.

Although conclusions from these studies, particularly whether or not ASICs are mechanosensors, are elusive, they provide clear evidence that ASICs are involved in mechanotransduction pathways. However, the question whether or not ASICs form mechanosensitive channels whose activity is directly influenced by mechanical forces remains unknown. By ‘direct influence’ it is meant that the mechanical force does directly induce conformational changes within the protein to affect the channel’s activity (mechano-electrical coupling).

The aim of this study was to focus on whether or not ASICs form mechanosensitive channels whose activity is influenced by mechanical force. To address this question human ASIC1_V3_, 2a and 3a subunits were expressed in *Xenopus* oocytes, an established cell model to study mechanotransduction of ion channels such as ENaC^26,27^, K^+^ channels^28^, CFTR^29^, TRP channels^30,31^ and P2X4 receptors^32^. Also ASIC3a was expressed in mammalian HEK-293 cells and exposed to SF. In all cases single ASIC subunits were expressed to form homomeric channels.

## Results

### Shear force at pH 7.4 has no effect on hASIC1_V3_, hASIC2a and hASIC3a activity

**Figure 1:**
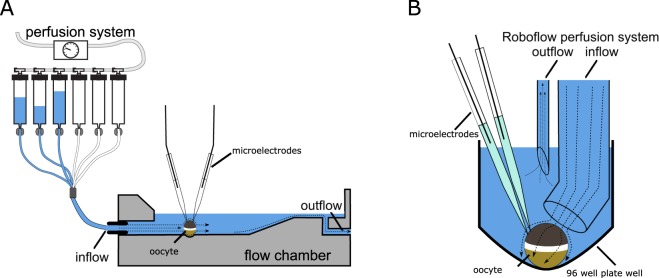
Illustration of SF application via two different systems. (**A**) Oocytes were placed in a custom made flow chamber where SF and acidic pH was applied at the same time via a pressurized perfusion system. (**B**) Oocytes were placed in 96 well plates where SF and acidic pH was applied via a tube directed at the oocyte.

**Figure 2:**
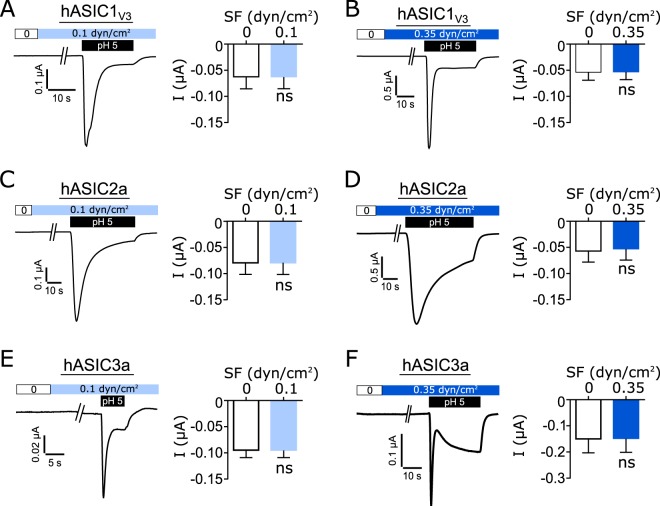
SF applied at pH 7.4 does not activate ASICs. Voltage-clamp experiments of ASIC expressing oocytes. Low (0.1 dyn/cm^2^) and high (0.35 dyn/cm^2^) SF rates were applied at normal pH conditions (pH 7.4) followed by pH 5 to confirm ASIC expression. Current trace and statistical analysis of hASIC1_V3_ at pH 7.4 when exposed from 0 to 0.1 dyn/cm^2^ SF (light blue) (**A**) and 0 to 0.35 dyn/cm^2^ (dark blue) (**B**). No change in transmembrane current was observed. The same results were observed for hASIC2a (**C**,**D**) and hASIC3a (**E**,**F**). Paired t test; ns: p > 0.05; n = 6–13.

SF-dependent activation of oocytes expressing human ASICs was analyzed by performing electrophysiological recordings. Oocytes expressing either hASIC1_V3_, hASIC2a or hASIC3a were placed into the recording chamber (Fig. 1) and exposed to SF at pH 7.4. Application of a SF rate of either 0.1 dyn/cm^2^ or 0.35 dyn/cm^2^ at pH 7.4 (switched from 0 dyn/cm^2^) did not induce any change in membrane current (Fig. 2). Expression and function of the channels was confirmed by subsequent application of pH 5, resulting in ASIC current characterized by a transient peak followed by a sustained current. These experiments indicate that SF applied at pH 7.4 does not affect the activity of expressed ASICs.

### Shear force combined with acidic pH increases the activity of ASICs

**Figure 3:**
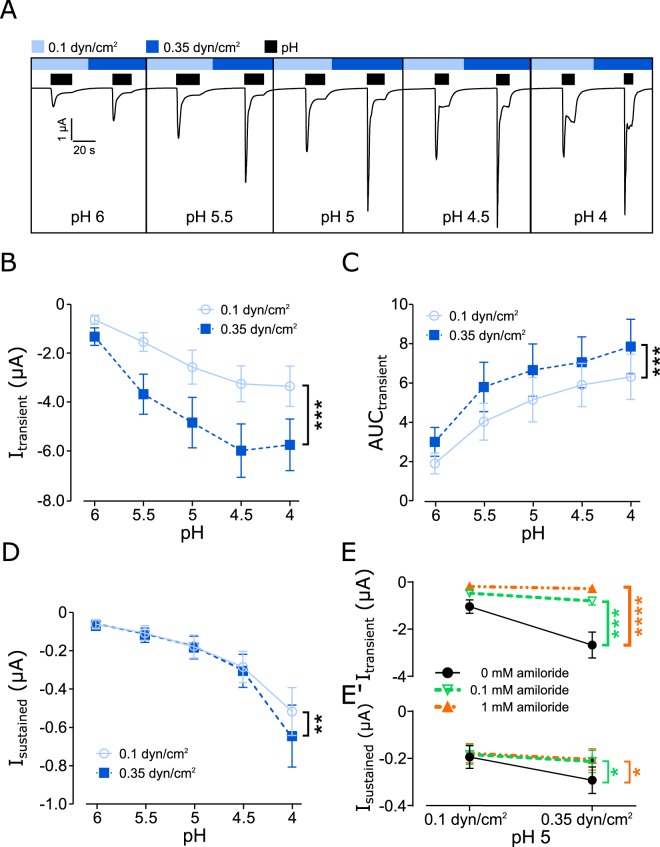
SF modulates the pH-induced activation of hASIC1_V3_. Oocytes were perfused with ORi (oocyte ringer’s solution) at pH 7.4 unless otherwise stated. (**A**) Representative current trace of hASIC1_V3_ being activated at acidic pH (application indicated by black bars) during low (0.1 dyn/cm^2^, light blue bar) and elevated (0.35 dyn/cm^2^, blue bar) SF conditions. (**B)** Summarized data of the pH-induced transient currents. (**C**) Analyses of the AUC and (**D**) the sustained currents. Transient (**E**) and sustained (**E’**) currents exhibited by pH 5 and SF are decreased by amiloride (0.1 and 1 mM). Two-way ANOVA; *p < 0.05; **p < 0.01; ***p < 0.001; n = 8–13.

In a new set of experiments, it was investigated whether ASICs activity can be modulated by SF, when SF was combined with acidic pH. Since the application of pH depends on perfusion, that will cause SF, it was decided to determine the pH-induced activation at low (0.1 dyn/cm^2^) and at elevated SF (0.35 dyn/cm^2^). This protocol was performed with pH 6, 5.5, 5, 4.5 and 4. In oocytes expressing hASIC1_V3_ the drop of pH activated a transmembrane current consisting of a large transient inward current followed by a smaller sustained current during prolonged acidification (Fig. 3A). This is in agreement with previously described pH-evoked currents for this human variant^33^. Washout with normal pH (7.4) returned the current back to baseline levels and it may be noted that the subsequent increase in SF from 0.1 to 0.35 dyn/cm^2^ did not induce any effects. However, when pH 6 was applied at 0.35 dyn/cm^2^ the transient current was almost doubled compared with the current observed at 0.1 dyn/cm^2^ (Fig. 3A). Repeating the protocol with pH 5.5 produced a similar result, the pH response was higher compared with the effect at pH 6, but the pH effect observed with 0.35 dyn/cm^2^ was again obviously larger compared with 0.1 dyn/cm^2^. This pattern (high current with elevated SF) was consistently observed for the remaining pH solutions (5, 4.5 and 4). Comparing the pH responses with low and elevated SF clearly shows that elevated SF significantly increases the transient current of hASIC1_V3_ (Fig. 3B).

In addition, the area under curve (AUC) and the sustained current during low and elevated SF was analyzed. The AUC (Fig. 3C) was determined for the transient current component and was significantly increased indicating that the effect with 0.35 dyn/cm^2^ does not only reflect changed channel kinetics. The increase AUC indicates an increase of net current in response to SF that may be caused either by an increased open probability or the additional activation of channels. Although the sustained current was also increased by SF, a pronounced effect was only observed at pH 4 (Fig. 3D), indicating that the sustained current plays a minor role for the SF response.

To confirm that the observed effects are hASIC1_V3_ mediated, amiloride (0.1 and 1 mM) was used to block pH 5 induced currents at low and high SF conditions (Fig. 3E,E’). Amiloride effectively blocked the pH 5 induced transient and sustained currents under low and high SF.

**Figure 4:**
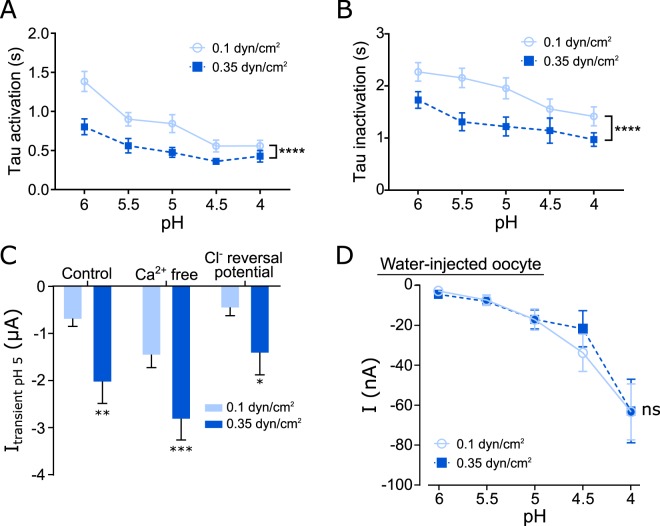
SF affects activation/inactivation kinetics and is ASIC specific. (**A**) Activation kinetics of acidic pH-induced currents in ASIC1_V3_ are faster with elevated SF. (**B**) Inactivation of pH-induced currents are faster with elevated SF. (**C**) Comparison of pH 5 evoked currents of hASIC1_V3_ injected oocytes were elevated at high SF (blue) compared to low SF (light blue) when incubated in Ca^2+^ free ORi or clamped at the calculated Cl^−^ reversal potential (−20 mV). (**D**) It may be noted that the small pH-induced currents observed with pH4.5 and 4 in water-injected oocytes are in agreement with previous reports^53^. However, these pH-induced responses were not influenced by SF. (**A,B,D**): two-way ANOVA ns: p > 0.05, ****p < 0.0001; (**C**) paired t test: *p < 0.05; **p < 0.01; ***p < 0.001; n ≥ 6.

The increased current in response to SF was accompanied by faster activation and inactivation times of the channel (Fig. 4A,B) indicating that the fast perfusion does result in a faster delivery of protons. The observed changed kinetics of the activation and inactivation between 0.1 and 0.35 dynes/cm^2^ were in the same range, indicating that this does not account for the increased current as quantified by the AUC.

To rule out the possibility that the observed effects are generated by endogenous Ca^2+^-activated Cl^−^ channels activated by the influx of Ca^2+^ through hASIC1_V3_, two different sets of experiments were performed. Firstly, SF effects at pH 5 of hASIC1_V3_ expressing oocytes were determined in absence of Ca^2+^ or secondly at a membrane voltage of −20 mV that corresponded approximately to the Cl^−^ reversal potential (Cl^−^ reversal potential was calculated via the Nernst equation using an intracellular Cl^−^ concentration of 44 mM (average from)^34^ (Fig. 4C). However, in the absence of Ca^2+^ as well as at a holding potential of −20 mV a robust SF-induced activation of hASIC1_V3_ was observed. Additional experiments with water-injected oocytes were performed to ensure that the observed SF effects are ASIC mediated and not due to other endogenous channels of the oocytes. Experiments as displayed in Fig. 3 were therefore repeated in water-injected oocytes (Fig. 4D). In these experiments small currents (approximately 100 fold smaller) were observed in response to acidic pH (e.g. pH 6, increase of 3 ± 1 nA; pH 4, increase of 64 ± 14 nA; n = 8). However, these currents were not affected by the application of elevated SF (Fig. 4D). Taken together, these observations indicate that SF increases the activity of hASIC1_V3_ when applied in combination with acidic pH and suggests a modulatory role of SF for the activity of ASIC1.

**Figure 5:**
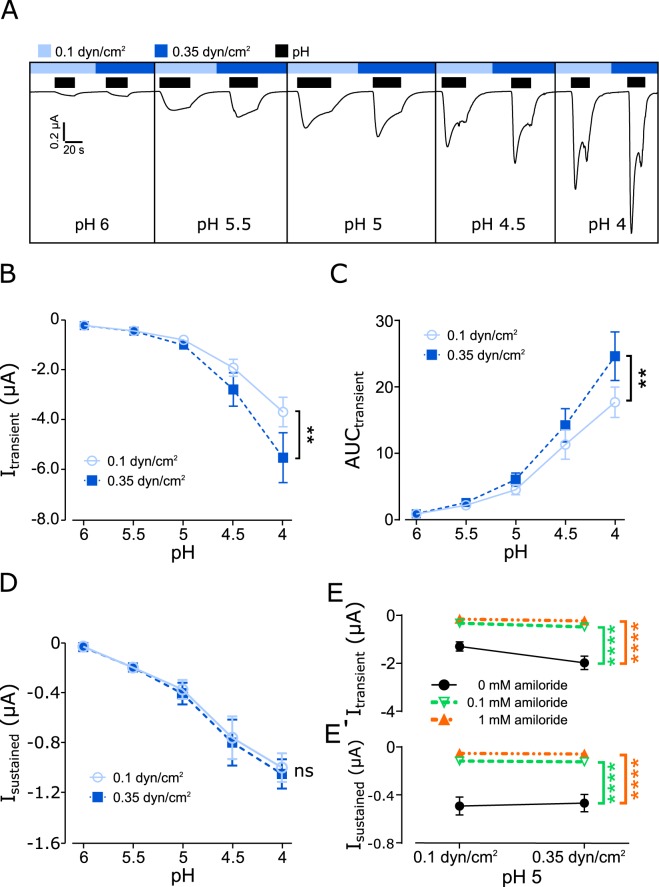
The activity of hASIC2a is modulated by SF. (**A**) Current trace of hASIC2a activity in response to acidic pH, during low (0.1 dyn/cm^2^) and elevated (0.35 dyn/cm^2^) SF. Comparison of the transient current (**B**), AUC (**C**) and the sustained current (**D**). Transient (**E**) and sustained (E’) ASIC2a currents evoked by pH 5 and SF is blocked by amiloride (0.1 and 1 mM). Two-way ANOVA; ns: p > 0.05; **p < 0.01; ****p < 0.0001; n = 8).

**Figure 6:**
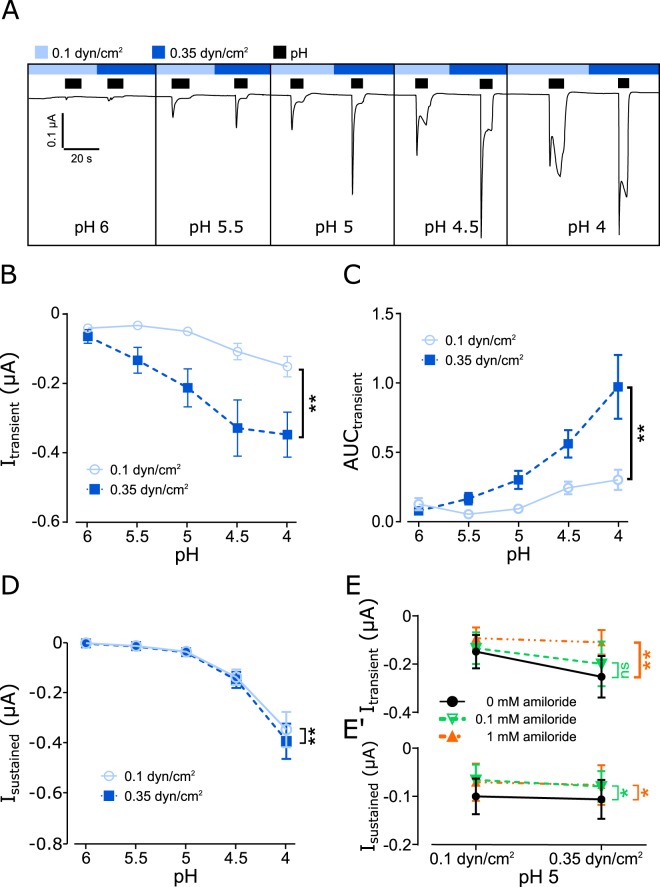
SF modulates the pH-induced activation of hASIC3a. (**A**) Current trace of hASIC3a being activated by acidic pH (black bars) during low (0.1 dyn/cm^2^) and elevated (0.35 dyn/cm^2^) SF conditions. Statistical quantification of the transient current (**B**) and AUC (**C**) during hASIC3a activation via different acidic pH and SF conditions. 0.35 dyn/cm^2^ SF leads to a stronger activation of hASIC3a compared to 0.1 dyn/cm^2^ under acidic pH conditions. (**D**) Statistical quantification of the sustained current of hASIC3a during different pH and SF conditions showed a significant increased sustained current when exposed to elevated SF (0.35 dyn/cm^2^) compared to low SF (0.1 dyn/cm^2^). (**E**,E’) hASIC3a currents evoked by pH 5 at low and high SF were blocked by amiloride. Two-way ANOVA analysis; *p < 0.05; **p < 0.01; n = 6–9.

Identical experiments were repeated with oocytes expressing either hASIC2a (Fig. 5) or hASIC3a (Fig. 6). Overall, for hASIC2a similar results were observed as with hASIC1_V3_. However, it may be noted that in some recordings with pH 6 and 5.5 no, or only small transient currents were detected (Fig. 5A,B). With pH ≤ 5 a transient current was consistently observed. Overall, the transient currents were increased with elevated SF compared with low SF. Accordingly, the AUC was significantly increased with high SF in comparison with low SF. The effect of SF on the sustained current of hASIC2a shows no differences when comparing effects at low and elevated SF (Fig. 5D). Similarly, to hASIC1_V3_, the pH 5 evoked currents were significantly blocked by amiloride at both high and low SF rates (Fig. 5E,E’).

**Figure 7:**
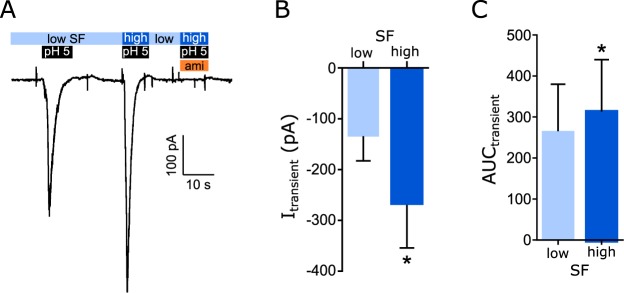
SF modulates the pH-induced activation of hASIC3a in HEK-293 cells. HEK-293 transfected with hASIC3a were perfused with pH 7.4 unless stated otherwise. A low (light blue) and high (blue) SF rate combined with pH 5 was applied via a tube directed at the cell. pH 5-induced currents (**A**,**B**) and AUC (**C**) were significantly increased with high SF compared with low SF. Additional application of 1 mM amiloride (orange bar) in the presence of pH 5 did not induce currents. Paired t test; *p < 0.05; n = 6.

For hASIC3a it was noted that the pH-induced currents were smaller compared with ASIC1_V3_ and ASIC2a indicating a reduced expression efficiency. Also with pH values ≤ 5 a slow activation of large sustained current was observed in contrast to hASIC1 and 2. Nevertheless, elevated SF increased the transient current and the AUC (Fig. 6B,C). As for hASIC1_V3_, changes of the sustained current in response to SF were detected although these were only clearly visible at pH 4. (Fig. 6D). The activation of hASIC3a by pH 5 and SF was also effectively inhibited by the application of amiloride (Fig. 6E,E’). The effect of SF on hASIC3a activity was also assessed in mammalian HEK-293 cells by whole-cell patch-clamp experiments (Fig. 7). Application of pH 5 activated transient currents that were significantly increased in presence of high SF compared with low SF (Fig. 7A,B). In addition, the AUC during pH 5 application was also increased during high SF application compared with low SF (Fig. 7C). Application of 1 mM amiloride blocked pH5-inducd currents suggesting that the observed effects were hASIC3a mediated.

### Shear force-induced activation of ASICs is consistent among different experimental setups

**Figure 8:**
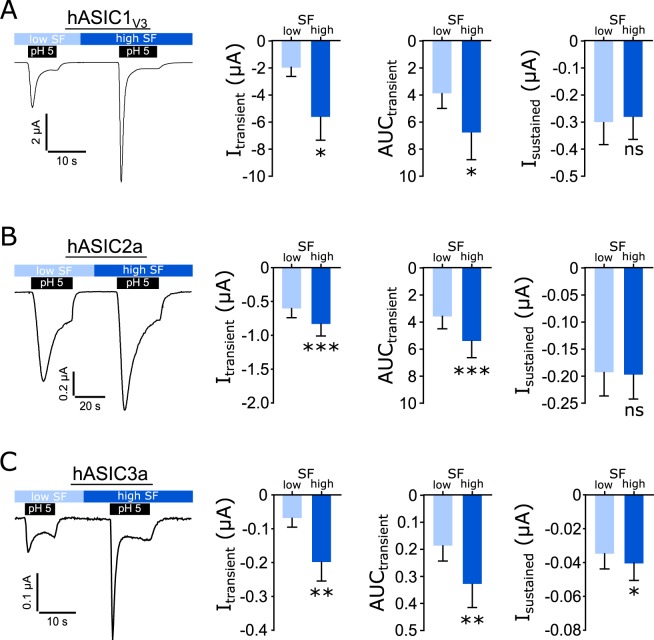
SF modulation of ASIC activity is reproducible in a different SF application system. Oocytes were perfused with ORi (pH 7.4) unless otherwise stated. A low and high SF rate combined with pH 5 was applied via a tube directed at the oocyte. The transient current and the AUC of hASIC1_V3_ (**A**) and hASIC2a (**B**) activation by pH 5 is significantly increased at the high SF rate. The sustained current was unaffected by different SF rates. (**C**) pH 5 applied at high SF significantly increased the transient and sustained current of hASIC3a activation as well as the AUC compared with low SF. Paired t test; ns: p > 0.05; *p < 0.05; **p < 0.01; ***p < 0.001; n = 7–11.

To further support our findings, we performed experiments using a new available automated setup to test the effect of SF on ASICs. Here SF is applied via a perfusion tube placed in close proximity to the oocytes that directed the applied fluid stream onto the oocyte (Fig. 1B). Experiments were performed at pH 5 with oocytes expressing hASIC1_V3_, hASIC2a or hASIC3a at a low and high SF rate (Fig. 8). For hASIC1_V3_ and 2a, pH 5 applied at high SF exhibited significantly increased transient currents and an increased AUC compared with low SF, whereas the sustained current remained unaffected. hASIC3a showed a significant increase for the transient and sustained current as well as for the AUC at high SF compared with low SF. These findings indicate that independent of the SF application system SF can increase the acidic pH response of hASIC1_V3_, -2a and -3a. Overall these findings provide strong support for a modulatory role of SF in regulating the activity of human hASIC1_V3_, hASIC2a and hASIC3a.

### Shear force activates ASICs in the presence of non-proton ligands at pH 7.4

**Figure 9:**
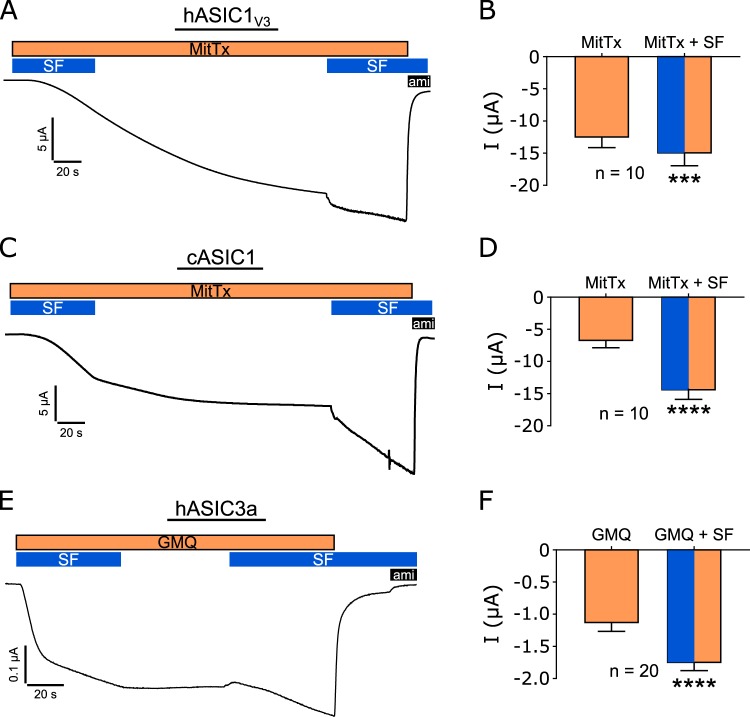
SF increases ASIC currents after pre-incubation with MitTx or GMQ at pH 7.4. Oocytes were perfused with ORi at pH 7.4 in absence or presence of MitTx (20 nM) or GMQ (1 mM). SF (0.35 dyn/cm^2^, blue bar) was applied via the perfusion system and initially used for 1 min to wash in MitTx or GMQ followed by a stop of the perfusion (no SF). After reaching a plateau, SF was applied again and the values before and after SF application were used for statistical analysis. 1 mM Amiloride (ami, black bar) was used to block ASIC mediated currents. Oocytes expressing hASIC1_V3_ (**A**,**B**) and cASIC1 (**C**,**D**) showed an increase in membrane current after the application of MitTx, which was even further increased by the additional application of SF. (**E**,**F)** Similar to MitTx, application of GMQ caused an activation of hASIC3a which was further increased by SF. Paired t test; ***p < 0.001; ****p < 0.0001; n = 10–20.

Non-proton ligands were identified to activate ASICs in a pH-independent way. In oocytes expressing hASIC1_V3_ (Fig. 9A) or cASIC1 (Fig. 9C), MitTx (20 nM) was washed into the well for approximately 1 minute and then the perfusion was stopped. This induced the continuous activation of a persistent current for another 3 min after the perfusion was stopped (Fig. 9A). The MitTx-induced current was not reversible by washout (data not shown). After stabilization of the MitTx-induced current the perfusion was turned on to apply SF (0.35 dyn/cm^2^). This resulted in a further significant increase of the current (Fig. 9B,D). The current was inhibited by amiloride (1 mM) indicating that the observed effects are ASIC mediated.

Identical experiments were performed with GMQ (1 mM) on hASIC3a expressing oocytes. Here, the GMQ-induced current plateaued within 1 and 2 minutes (Fig. 9E). The subsequent application of SF in the presence of GMQ also resulted in a further increase of the measured current. In contrast to MitTx the removal of GMQ from the bath resulted in a reduction of the current. Amiloride was applied at the end of the experiment.

Neither MitTx nor GMQ had any effects in water-injected oocytes (data not shown) suggesting that the observed effects with either compound are ASIC specific. These experiments further support the ability of shear force to modulate ASIC activity that in this case is pH-independent.

## Discussion

Results in this study indicate that mechanical force, which was applied in the form of SF via a fluid stream, modulates the activity of heterologously expressed ASIC channels. Growing evidence over the last decade revealed that ASICs are involved in mechanotransduction pathways. This evidence was obtained from studies using various transgenic animal models, primarily assessing animal behaviour in response to different mechanical stimuli^19,25^. Some of the animal studies provided contradictory results, making identification of the role of ASICs in mechanotransduction pathways challenging. In ASIC3 null mice, for example, a decreased sensitivity to a noxious pitch was reported^35^ whereas others reported an increased sensitivity in response to the tail pressure test^36^. Leaving these contradictory observations aside, it is undisputed that ASICs are involved in mechanotransduction pathways. However, whether ASICs are directly involved in the sensation and transduction of mechanical forces or solely involved ‘downstream’ of the transduction processes remains uncertain from these studies.

Previous articles defined criteria for channels to function as ‘mechano-electrical channels’^37^. One criterion proposes that putative mechanosensitive channels should recapitulate properties of native currents when heterologously expressed, including activation through known agonists^37^. To our knowledge whether ASICs constitute ‘mechano-electrical channels’ when heterologous expressed is unknown^38^ and was the major focus of the present study. Thus the main question of this study was whether or not heterologously expressed ASICs are activated by mechanical force.

Expression of human ASIC1_V3_, ASIC2a, ASIC3a and chicken ASIC1 in *Xenopus* oocytes and their exposure to SF revealed three main findings. (1) At physiological pH (7.4), no ASIC currents were measured in response to SF. This indicates that the channels do not respond to mechanical force per se. (2) Exposing the channels to elevated SF in combination with acidic pH resulted in increased currents when compared with low SF. (3) Pre-activation of ASICs with non-proton ligands (MitTx and GMQ) at pH 7.4 provided SF-induced currents. These findings suggest that SF does modulate the activity of ASICs.

Strong support for a role of SF to modulate ASICs activity derives from the observations made with hASIC1_V3_ (Fig. 3A). Here the pH response with pH 4.5 and 4 at 0.1 dyn/cm^2^ did not further increase compared with the effect at pH 5. This indicates maximal pH-dependent activation of hASIC1_V3_ at pH 5 as previously shown^39^. Astonishingly, the effect that was observed with elevated SF increased the pH response by almost 100%. This SF-dependent activation of the transient current (at pH 5) was not achieved with pH 4.5 or pH 4 – which represents a considerable increase in proton concentration. It is unlikely that the SF effect can be reasoned solely by the kinetic of proton delivery to the channel. The change in the channel activation kinetic with elevated SF is similar to the inactivation kinetic. This may result in an increased transient current amplitude with elevated SF, but it is unlikely to affect the AUC as a measure for overall increased current. These two observations imply two things: (1) SF affects the channel conformation on top of pH and (2) the force has a direct modulatory effect on channel gating, likely by increasing the open probability of pH-‘pre-activated’ channels.

Activation of ASICs by acidic pH alone usually causes a strong transient current, which desensitizes during prolonged acidification followed by a smaller sustained current that does not desensitize^6^. The sustained current exhibits different biophysical and pharmacological characteristics compared to the transient current and the reason for this phenomenon is not clear^40–42^. Although we observed increased transient and sustained (except for hASIC2a) currents in response to elevated SF, the effect on the transient currents was much more pronounced. This finding is in agreement with two different conformations of the channel pore that are responsible for the transient and sustained current^6^, indicating different gating mechanisms. The SF effect on the transient current is also in agreement with findings that the extracellular domain is mainly involved in mediating the transient current^43–45^, whereas transmembrane and intracellular domains mainly determine the characteristics of the sustained current^42,46^. The importance of the transient current for the SF effect is also supported by the results obtained with hASIC2a. Here the SF effect was obviously increased in conjunction with an overall increasing transient current and no change of the sustained current was observed. This supports the putative role of the large extracellular domains as force sensors within the ENaC/degenerin protein family^47,48^.

Studies have identified ASIC ligands, such as MitTx and GMQ which cause a persistent activation of the channels^12^. Subsequent studies identified GMQ to modulate the pH activation of various ASICs but only with ASIC3 a persistent current was described^14^ that is in accordance with previous and our observations. There is also evidence that introducing cysteines combined with the application of sulfhydryl reactive compounds activated persistent ASIC1 current at pH 7.4^49^. These studies indicate that ASICs can be activated and operate in a pH-independent way. The use of non-proton ligands in our study allowed to study the effect of SF on ASIC activity at physiological pH, to assess whether the SF effects observed with low pH are due to the increased velocity of delivering protons in combination with the activating/inactivating kinetics of the channels in response to low pH.

Apparently, these non-proton ligands ‘switch’ the channels in a constitutively active operation mode that is a characteristic feature for related channels like ENaC. The SF-mediated responses of hASIC1 and 3 were similar to that of ENaC^26,27^. Although the gating mechanism in response to non-proton ligands might be different compared with the gating mechanism at low pH, these data further support the suggestion that SF modulates ASIC activity.

To our knowledge, this study showed for the first time that the activity of heterologously expressed ASICs is modulated by mechanical force. Although the results obtained may not translate into the physiological situation, they provide further evidence to support the role of ASICs as mechanosensors. The results can explain ASIC’s role in tissues such as renal tubular epithelial cells^50^, arterial baroreceptor^21^ and cerebral arteries^51^, where the flow of urine/blood causes SF and local changes of pH might occur. In addition, it may be speculated that the SF response may also account for the perception of touch and pain in response to touch. Touch-induced deformation of tissues and cells can result in shear movements at the cell surface that may be sensed by the extracellular domain of the channel and contributes to their activation.

If ASICs have a role as mechanosensors it seems to depend on acidic pH environments and/or the presence of non-proton ligands. The pH used in our study were quite acidic implying that pH is not a major regulatory mechanism for mechanical modulation of ASICs. Nevertheless, tissue acidification and the release of GMQ related compounds such as agmatine can occur during inflammation^13^. Thus, local environments defined by elevated proton concentrations in combination with inflammatory compounds and venoms could render ASICs to become mechanosensitive. The importance of local environments for ASIC function is further supported by evidence that arachidonic acid, as part of the membrane lipid environment, activated ASIC3 at pH 7.4^11^. Thus ‘pre-conditioning’ defined by specific local environments (e.g. pH and inflammatory mediators) could be required for mechanical activation. The combination of these factors in local environments – or the lack of – could explain existing inconsistent reports whether or not ASICs are mechanosensitive channels when comparing results from *in vitro* studies with results from transgenic animal models.

It will require future investigations to further reveal the role of SF for ASIC activation. Accordingly, future studies may also reveal whether pH, lipids or non-proton ligands would be more important as pre-conditioning factor for the mechanical activation of ASICs. It can also not be excluded, that all three factors can influence mechanical activation interdependently, which may further complicate identification of the underlying mechanism.

It is obvious that our finding does not provide a clear-cut answer to the question whether or not ASICs are mechanosensitive since this may primarily depend on the definition used to define what a mechanosensitive channel is. Although being aware that this may be disputed in the field, we propose that our observations justify to consider ASICs as being mechanosensitive channels.

## Materials and Methods

### Ethics statement

Lab bred female *Xenopus laevis* were supplied through NASCO (Fort Atkinson, USA) and used for oocyte extraction via surgery. Animals were housed in a XenoPlus housing system (Tecniplast, Sydney, Australia). All procedures were approved by the University of Otago Animal Ethics Committee (approval numbers: 114/13 and 83/16) and conducted in accordance with the New Zealand Animal Welfare Act.

### Oocyte isolation from adult females *Xenopus laevis*

Female South African clawed frogs (*Xenopus laevis*) were anaesthetized with 1.3 g/L MS-222 (Ethyl 3-aminobenzoate methanesulfonate; Sigma-Aldrich, USA). By a small incision through the skin and muscle layer of the abdomen, a small part of the ovaries was removed and stored in cultivation oocyte Ringer’s solution (CulORi) containing (in mM): 90 NaCl, 1 KCl, 2 CaCl_2_, 5 HEPES, 2.5 Na^+^-pyruvate, 0.06 penicillin, 0.02 streptomycin; plus 50 µg/mL tetracycline, 100 µg/mL amikacin, 100 µg/mL ciprofloxacin, pH 7.4. After removal of the oocytes, the incision was closed in two layers by suturing the muscle and skin layers separately. Animals were then recovered in a separate tank (body submerged and the head above water to prevent drowning) and consistently monitored for up to an hour. After this, animals were placed in a separate tank within the housing system and monitored daily for 5 days. Thereafter animals were placed back into their original tank.

### Heterologous expression of ASICs

In preparation for expression in oocytes, the ovaries were dissected in small pieces by forceps and then incubated (approximately 90 min) in collagenase-containing CulORi solution (1.5 mg/mL). This was followed by a subsequent incubation (10 min) in Ca^2+^-free oocyte Ringer’s solution (composition in mM: 90 NaCl, 5 HEPES, 1 KCl, 1 EGTA, pH 7.4). Stage V-VI oocytes were isolated for cRNA-injection. cRNA (2 ng/oocyte) encoding the human ASIC1 variant3 (NCBI#: NM_001256830.1), human ASIC2a (NCBI#: NM_001094.4), human ASIC3a (NCBI#: NM_004769.3) and chicken ASIC1 (NCBI#: NM_001040467.1; 0.2 ng/oocyte) was injected into the oocytes via microinjection using a nanoject II Auto-Nanoliter Injector (Drummond Scientific Company, Broomall, USA) or Roboinject (Multichannel Systems, Reutlingen, Germany). Control oocytes were injected with corresponding volumes of nuclease-free water. After injection, oocytes were cultured individually (96-well plate) in a low-Na^+^-solution (composition in mM: 10 NaCl, 80 NMDG (N-methyl-D-glucamine), 1 KCl, 2 CaCl_2_, 5 HEPES, 2.5 Na^+^-pyruvate, 0.06 penicillin, 0.02 mM streptomycin, 50 µg/mL tetracycline, 100 µg/mL amikacin, 100 µg/mL ciprofloxacin, pH 7.4) at 17 °C. Two-electrode voltage-clamp (TEVC)-recordings were performed at room temperature within 64–74 hours post injection.

HEK-293 cells (German Collection of Microorganisms and Cell Cultures (Braunschweig, Germany) were cultured in DMEM media (Biochrome, Berlin, Germany) supplemented with 4 mM L-glutamine and 10% foetal calf serum (Biochrome) and 2 mM sodium pyruvate. Cells were transiently transfected with the cDNA of hASIC3a (pcDNA3.1 vector) and EGFP (pcDNA3.1 vector) using FuGene 6 transfection reagent (Roche, Mannheim, Germany) according to the manufactures protocol. Transfected cells were maintained for 24 h in an incubator (37 °C and 5% CO_2_). Subsequently, cells were seeded on plastic petri dishes and incubated for another 4 h before patch-clamp experiments were performed on cells positive for EGFP.

### Two-electrode voltage-clamp experiments

The oocytes were placed in a custom-made flow chamber and perfused with oocyte Ringer’s solution (ORi, containing in mM: 90 NaCl, 1 KCl, 2 CaCl_2_, 5 HEPES, 5 MES, pH 7.4) driven by a pressure regulated perfusion system (ALA Scientific Instruments, New York, USA). The flow-chamber had a channel for perfusion, designed to allow a consistent application of SF to the oocyte´s surface and a rapid solution-exchange (Fig. 1A). The flow rates were adjusted to 1.6 mL/min and 3.2 mL/min. Considering the geometry of the flow channel, these flow rates were estimated to correspond to approximately 0.1 and 0.35 dyn/cm^2^ of SF on the oocytes surface. Shear force rates were calculated as described before (Althaus *et al*. 2007). To measure transmembrane currents, the oocytes were clamped at a membrane potential of –60 mV using a TURBO TEC-05 amplifier (NPI, Tamm, Germany). The currents were digitized via a PowerLab 4/35 and LabChart (ADInstruments, Dunedin, New Zealand). Activation of ASICs was induced by the application of low pH ORi (pH adjusted with NaOH to 6, 5.5, 5, 4.5 and 4) at 0.1 dyn/cm^2^ or 0.35 dyn/cm^2^ of SF.

Some experiments were performed using an automated TEVC system (Roboocyte2, Multichannel Systems, Reutlingen, Germany) utilizing a 96 well plate design for holding and recording of oocytes. For the application of SF, the Roboocyte2 perfusion tube was modified to enable the application of SF (Fig. 1B). Therefore, a perfusion tube (glass capillary with 1.2 mm internal diameter) was heated and slightly bent to direct the fluid stream at the oocyte similar to an approach used before (Fig. 1B and^27^). The perfusion rates for SF application were adjusted via the software and administered through the perfusion pumps of the system. The flow rates were adjusted to 2.8 mL/min and 4.8 mL/min and this resulted in estimated SF rates of 0.1 and 0.35 dyn/cm^2^ that produced ASIC responses that were similar to those observed with the flow-chamber.

Amiloride was used as a pore blocker of ASICs^52^ in concentrations of 0.1 and 1 mM (dissolved in ORi) to block ASIC currents. The peptide component MitTx (20 nM, Alomone Labs, Jerusalem, Israel) from the Texas coral snake, *Micrurus tener tener* and GMQ (1 mM, Sigma-Aldrich) were used as non-proton ligands to activate hASIC1_V3_ and cASIC1^12^ as well as hASIC3a^13^ respectively.

### Whole-cell patch-clamp experiments

Whole-cell recordings were performed using an Axopatch 200B amplifier and were digitised via a Digidata 1440 A. Data was recorded on a personal computer with Clampex 10.7 and analysed using Clampfit 10.7 (Axon Instruments, Foster City, USA). The standard bath solution contained (in mM) 140 NaCl, 1.2 MgCl_2_, 1.2 CaCl_2_, 5 KCl, 10 HEPES, pH 7.4 or pH 5. The pipette solution contained (in mM) 145 CsCl, 8 NaCl, 2 MgCl_2_, 10 HEPES, pH 7.2). Rapid solution exchange and SF application (0.1 mL/min and 0.2 mL/min) was performed via an RSC-200 perfusion system (Bio-Logic Science Instruments, Seyssinet-Pariset, France) with the perfusion tube placed in close proximity of the patched cell. Patch pipettes were made of borosilicate glass (Hilgenberg, Malsfeld, Germany) and had tip resistances between 2 and 4 MΩ. A gap-free acquisition mode was used with analogous filtering at 2 kHz. Analysis was performed after additional filtering at 100 Hz.

### Data analysis

Data are expressed as mean ± standard error of the mean (SEM). Numbers of experiments are presented as *n*. Oocytes from at least two animals were harvested and used for each experiment. Values from the electrophysiological recordings were analyzed with GraphPad Prism 6.07. Tau values as a measure for channel activation/inactivation kinetics were determined by measuring the time required to reach 63.2% of the transient current amplitude, respectively the time required to decline to 36.8% from the maximum current. Statistical comparisons were made using two-way ANOVA (Grouped analysis) or paired Student’s t-test (indicated with data presentation). Statistical differences were indicated as following ns: p ≥ 0.05; *p < 0.05; **p < 0.01; ***p < 0.001; ****p < 0.0001.

## Data Availability

The datasets generated during and/or analysed during the current study are available from the corresponding author on reasonable request.
